# A data driven comparison of hybrid machine learning techniques for soil moisture modeling using remote sensing imagery

**DOI:** 10.1038/s41598-025-27225-0

**Published:** 2025-12-04

**Authors:** Prabhavathy Settu, Mangayarkarasi Ramaiah

**Affiliations:** School of Computer Science Engineering and Information Systems, Vellore Institute of Technology, Vellore, 632014 India

**Keywords:** Soil moisture, Machine learning, XGBoost, Random forest, Rainfall, Hydrology, Natural hazards

## Abstract

Soil moisture plays a very important role in agricultural production, water and ecosystem well-being particularly in rain-fed areas such as Tamil Nadu, India. This study evaluates and compares the performance of eleven machine learning models, Linear Regression (LR), Support Vector Machine (SVM), Random Forest (RF), Gradient Boosting (GB), XGBoost (XGB), Artificial Neural Network (ANN), Long Short-Term Memory tuned with Ant Lion Optimizer (LSTM-ALO), LSTM optimized with the weighted mean of vectors optimizer (LSTM-INFO), Random Vector Functional Link optimized using Enhanced Reptile Optimization Algorithm (RVFL-EROA), Artificial Neural Network optimized via Elite Reptile Updating Network (ANN-ERUN), and Relevance Vector Machine tuned with Improved Manta-Ray Foraging Optimization (RVM-IMRFO) for predicting monsoon-season soil moisture using rainfall and topographic parameters (slope, aspect, and Digital Elevation Model (DEM)). The models were trained using rainfall data from the India Meteorological Department (IMD) and high-resolution soil moisture datasets. Model performance was assessed using Root Mean Square Error (RMSE), Mean Absolute Error (MAE), Nash–Sutcliffe Efficiency (NSE), Kling–Gupta Efficiency (KGE), and Combined Accuracy (CA). Among all models, XGBoost and Random Forest achieved the highest accuracy (RMSE = 0.018–0.019 m³/m³; NSE ≈ 0.983–0.984; KGE ≈ 0.988), followed closely by ANN and ANN-ERUN (RMSE ≈ 0.020 m³/m³; NSE ≈ 0.980). The hybrid models RVFL-EROA and RVM-IMRFO demonstrated moderate performance (RMSE = 0.045–0.052 m³/m³; NSE = 0.87–0.90), while LSTM-ALO and LSTM-INFO performed relatively lower due to optimizer sensitivity and data non-stationarity. Error distribution and scatter plots confirmed that ensemble and metaheuristic-enhanced models effectively captured the non-linear soil moisture variability in topographically diverse regions. This evidence shows that ANN-ERUN, RVFL-EROA and RVM-IMRFO as hybrid metaheuristic learning methods can be used to complement ensemble models like XGBoost and Random Forest to estimate soil moisture in data-sparse, heterogeneous landscapes. Higher-level hybrid tuning strategies and longer-term models should be investigated in future research in an effort to promote predictive robustness.

## Introduction

Soil moisture is the amount of water that exists within the dry area of the soil, which is mostly, in the root zone, and is of critical importance in many environmental, agricultural, and hydrological processes^1^. Dynamics of soil moisture are determined by several processes, which include precipitation, evapotranspiration, infiltration, and percolation that are controlled by the soil texture, structure, vegetation cover, and climatic conditions^2^. These processes lead to a constant change of the moisture content of different depths in the soil^3^. The soil moisture distribution has a major impact on the local and global hydrological cycles, which add to the energy flux, carbon sequestration, and weather patterns^4^. Soil moisture in agricultural practice is a crucial aspect in defining crop health and crop productivity^5^. The presence of sufficient moisture in the soil promotes favorable growth, whereas its absence may result in lower yields or even loss of crops, making the soil dry up ^6^. Soil moisture in hydrology applications determine the quantities of rainfall that are absorbed into the soil or those that are carried off as surface runoff and this adds to the chances of flooding^7^. Long-term soil moisture data is used in climate studies for weather forecasting and investigation of climate change effects on water availability, vegetation, and extreme weather patterns (such as floods and droughts)^8^. In the regard of disasters, elevated soil moisture may result in soil structure that is more vulnerable to landslides in hilly and mountainous areas^9^. Low levels of soil moisture, especially during dry seasons, on the other hand pose a high risk of forest fires^10^. Since soil moisture is critical as a component of the environmental and agricultural systems, the proper mapping of soil moisture and continuous observation of this aspect have become crucial. Conventional point scale measurements of soil moisture with in-situ sensors are very accurate but have a low spatial resolution^1^. The difference between soils of different landscapes, as well as vegetation types, topography, and weather conditions, brings out the importance of high-resolution spatial coverage; subsequent continuous monitoring of soil moisture also contributes to the formation of its temporal dynamics, valuable in real-time applications, such as weather forecasting, irrigation management, and disaster response^11^.

Conventionally, measurement of soil moisture has been conducted via in-situ measurement, and this is precise, but time-consuming, labour intensive, and only gives point based measurements of soil moisture^12^. Gravimetric method, which measures the moisture content of soil by weighing the soils prior to and after drying them in the oven, is one of the most precise methods in existence but has the disadvantage of being labor intensive and high scaling is very limited, which can be applied when measuring large scale monitoring^13^. Time Domain Reflectometry (TDR) and neutron probes are continuous monitors at a particular location, which are expensive and cannot provide coverage over a wide area due to their cost and small coverage^14^. Moreover, these techniques usually tend to measure moisture only at shallow levels, and are not able to capture the spatial variability of soil moisture in heterogeneous landscapes. Soil moisture measurement has developed over the last few decades with the increasing popularity of remote sensing technology^15,16^. Remote sensing provides the capability to observe soil moisture on a large scale, which is no longer limited by the space constraints of in situ techniques^17,18^. Missions such as SMAP (Soil Moisture Active Passive) and AMSR-E (Advanced Microwave Scanning Radiometer for EOS), use passive remote sensing to measure natural microwave emissions emitted by the soil surface and affect the soil moisture levels^19^. Even though the passive sensors offer extensive global coverage, their spatial resolution is usually low (between 25 km and 40 km) and hence they cannot be useful in applications that require high spatial resolution, like precision agriculture^20^. Active remote sensing (Synthetic Aperture Radar) on the other hand emits microwave pulses and records the back scatter of the earth surface. SAR sensors such as Sentinel-1 have a better spatial resolution (10 m) and can be used in all weather conditions, which is especially advantageous when it comes to measuring the soil moisture under the clouds^17,21^.

The past three decades have witnessed an exceptional change in soil moisture surveillance, which is influenced by satellite missions and computing innovations. The SSM/I (Special sensor microwave/ imager), AMSR-E and SMAP satellite missions enhanced the spatial and temporal resolutions of soil moisture. Other missions like Sentinel-1 offered better resolution data (6–12 days, with higher resolution (~ 10 m) depending on the region^22^. These missions have provided the foundations of soil moisture mapping at a higher level of quality and space and time coverage^15^. Majority of space missions can only get soil moisture on the surface of the top few centimetres of soil which might not reflect the actual moisture content in the soil at root depth, which is of essence in terms of agriculture^23^. Optical and thermal techniques, especially, are constrained by cloud cover and can only give estimates of surface soils moisture^24^. Also, the data obtained through remote sensing are prone to environmental disturbance, including vegetation cover, surface roughness and atmospheric conditions, that can skew the accuracy of soil moisture^16^.

Based on these constraints, there has been increased interest in merging datasets retrieved by space missions with other datasets, e.g., rainfall, to enhance the spatial and temporal quality of soil moisture estimations. One of the major causes of soil moisture variation is rainfall and most hydrological models like the SWAT (Soil and water assessment tool) and VIC (Variable infiltration capacity) model utilize rainfall as an input to model the dynamics of soil moisture^25,26^. Nevertheless, these classical models tend to be empirical in nature and need a great amount of parameterization, which constrains their versatility and generalization to different circumstances. The machine learning (ML) approaches present an effective alternative, where data-oriented modeling of soil moisture is possible based on the rainfall and additional environmental variables.

Random Forests (RF), Artificial Neural Networks (ANN), and Support Vector Machines (SVM) ML models have been used to predict soil moisture using rainfall data, temperature, and satellite-acquired such indices as the Normalized Difference Vegetation Index (NDVI)^27–30^. These models can represent hard to understand non-linear correlations between the input variables making them able to predict soil moisture with greater accuracy, particularly in areas with limited data. For instance, research has demonstrated that combining rainfall datasets (including CHIRPS (Climate Hazards Group InfraRed Precipitation with Station data) and TRMM (Tropical Rainfall Measuring Mission)) with machine learning models can help to increase the temporal scale of soil moisture predictions, making daily (or even hourly) predictions with narrower spatial scales. This method is especially useful in places where there are no or limited satellite derived soil moisture measurements because of cloud cover or low sensor resolution. Recent research has used traditional neural networks together with meta heuristic optimizers to improve forecasting. For instance, the accuracy of runoff and temperature prediction by LSTM networks that are trained with the Ant Lion Optimizer (LSTM-ALO) and with an information-driven algorithm (LSTM-INFO) has been enhanced^31,32^. Similarly, Random Vector Functional Link models trained with improved Reptile/Remora has been demonstrated to perform well on environmental time-series tasks in both Random Vector Functional Link (RVFL-EROA) and neural networks trained with an elite-reptile updating (Enhanced Runge Kutta) algorithm (ANN-ERUN)^33,34^. Furthermore, relevance vectors machines that are optimized with a Manta-Ray Foraging Optimizer (RVM-IMRFO) have been successfully used in pan evaporation and hydrology forecasting^34^.

The application of ML methods to estimate soil moisture is a significant paradigm change in the way we treat this important environmental variable. ML models have the potential to combine the data of various sources such as space-based, as well as ground-based measurements, and weather data to generate better and high-resolution soil moisture maps. Machine learning can overcome the structural limitations of conventional methods of soil moisture measurements by utilizing the high-tempor and high-spatial quality of rainfall measurements and the spatial granularity of remote sensing. Moreover, the global and regional applications are appropriate because of the scalability of ML models, which can be used to make real-time predictions and long-term observability.

This paper is a new machine learning model to estimate soil moisture in the hydro-climatically diverse and monsoon-dependent Tamil Nadu region of India. The difference is that high-resolution topographical data (slope, aspect, and DEM) have been integrated with rainfall data provided by the IMD to predict soil moisture with a better spatial-temporal resolution. Rainfall is one of the major sources of soil moisture and thus the study utilizes ML, including RF, SVM, GB, XGBoost, LSTM-ALO, LSTM-INFO, RVFL-EROA, ANN-ERUN, and RVM-IMRFO to predict soil moisture. These environmental variables were aimed at improving the spatial and temporal accuracy of soil moisture predictions as they play an important role in determining the distributions of soil moisture. The study supports the more general discipline in demonstrating that data-driven approaches can complement the forecasting and tracking of soil moisture, which are limitations of conventional methods of measurement.

### Study area

Tamil Nadu is situated at the extreme south of Indian subcontinent with the Bay of Bengal on the east, Kerala on the west, Karnataka on the northwest, and Andhra Pradesh on the north. The geographical coordinates of the state vary about 8° to 13.75° North latitude and 76° to 80.5° East longitude. It spans approximately 1,30,058 square kilometers, and it is one of the populated and most productive states in India. The topography of Tamil Nadu is highly diverse and the land of this state is made up of coastal lands, mountain ranges, plateaus, and river basins. The eastern plains are largely flat and alluvial with a long coastline of 1,076 km and the Western Ghats to the west with a height of over 2,500 m that is very vital in shaping weather patterns and distribution of rainfall.

**Fig. 1:**
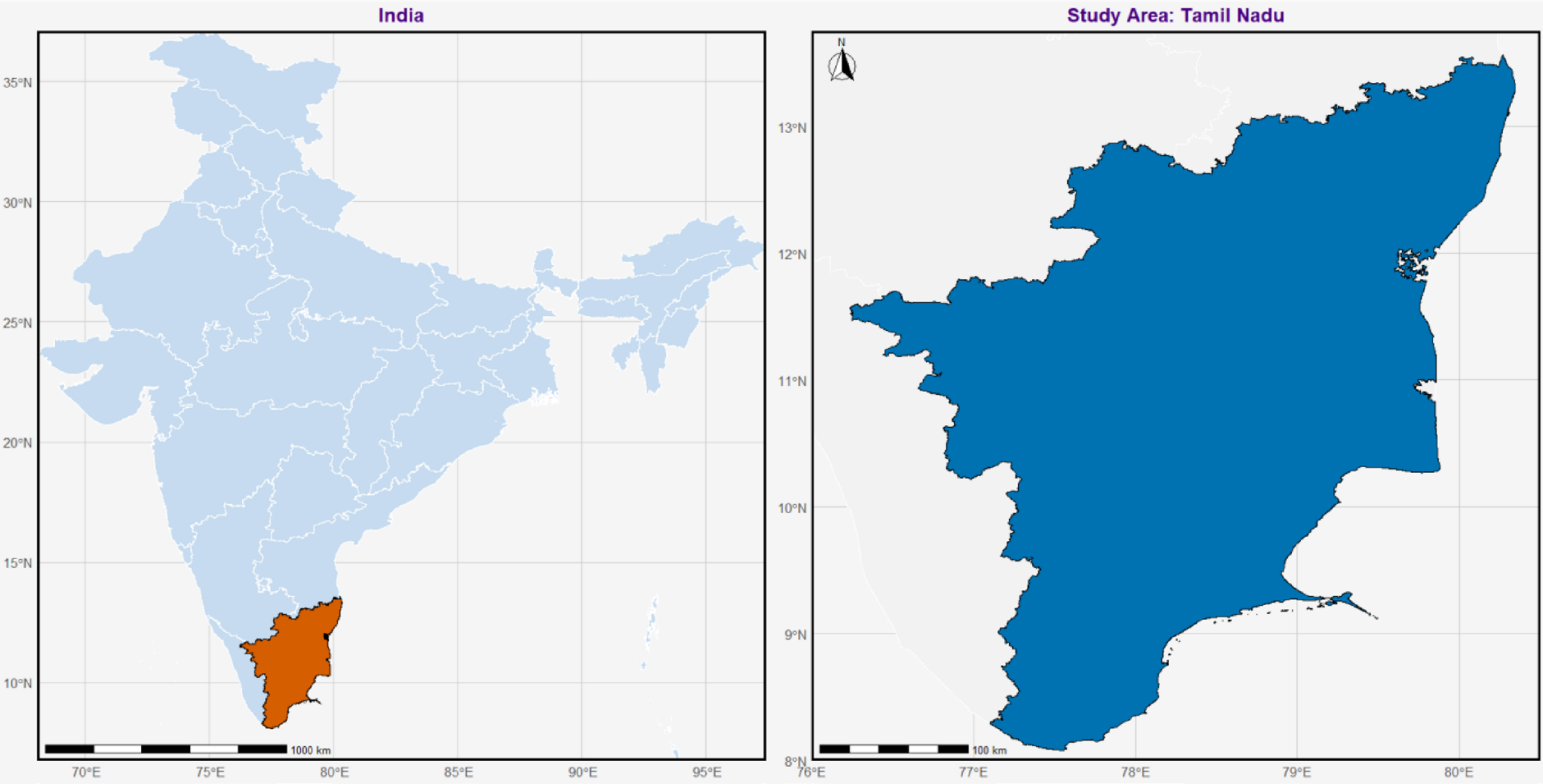
Geographical location of the study area.

The heart of the state is characterized by the Deccan Plateau which consists of rolling uplands, rocky outcrops and mixed soil types leading to the fact that the soil moisture retention capacity varies. The Cauvery, Vaigai and Thamirabarani form the major river systems in Tamil Nadu and they comprise fertile river basins and the Cauvery delta is among the most agriculturally productive area in the northeast. Tamil Nadu has climatic conditions that are tropical in nature, with its main seasonal climates being southwest monsoon between June and September, northeast monsoon between October and December and dry season between January and May. The northeast monsoon is the major source of rainfall in the state being the main recipient in the eastern coastal plains and river basins and the central and western areas are less favored to receive the rain and therefore reliant more on irrigation. An average of 945 mm of rainfall falls on Tamil Nadu annually though this is not evenly spread because of the intricate topography of the state. Geographical location of the study area is presented in Fig. 1.

### Datasets

Various datasets were used in this research to examine the correlation between rainfall, topography, and soil moisture in Tamil Nadu. The datasets to be used in the current study are SRTM (Shuttle Radar Topography Mission) DEM, Slope, Aspect, IMD Rainfall Data, and High-resolution Gridded Soil Moisture Data obtained in a prior study 35. Nayak et al. 2018 developed the soil moisture data set with Land Data Assimilation System (LDAS) that incorporates forcing data on various sources such as MERRA (Modern-Era Retrospective analysis for Research and Applications), MERRA2 (version 2), CFSR (Climate Forecast System Reanalysis) and ERA-Interim (European Centre for Medium-Range Weather Forecasts Interim Reanalysis), GLDAS (Global Land Data Assimilation System). The soil moisture information obtained by this approach is within the range of 2001 to 2014 during the monsoon months (June to September). The soil moisture data was being provided at a spatial resolution of 4 km, and thus, it was also resampled to the IMD rainfall data, which was provided at a resolution of 0.25 degrees to maintain uniformity across datasets in the analysis.

## Methodology

There were also a number of preprocessing that had to be done before the data could be integrated and compared. First, soil moisture data of initial resolution 4 km was resampled on a 0.25-degree grid with bilinear interpolation. This resampling guaranteed the consistency in spatial resolution, which allowed a direct comparison between the IMD rainfall data and the resampled one. In addition, topographical parameters like slope, and aspect were also obtained using SRTM DEM. Slope, which measures the steepness of the slope, was determined to determine its impact on water runoff and water infiltration. The aspect which is dependant on the orientation of the slopes was also calculated, since it influences the exposure to solar radiation and, therefore, the evapotranspiration rates that influence the retention of moisture in the soil.

This research concentrated its attention on monsoon seasons, that is, June to September, years 2001 to 2014. This time synchronization played an important role since these are the most important rainy months in Tamil Nadu and these months affect the agricultural activities and water management. The rainfall data and the soil moisture data were filtered to only these months to allow a complete examination of the seasonal influence of soil moisture dynamics. The temporal synchrony between the datasets is used to clarify the correlation between the trends in rainfall and soil moisture at a critical monsoon season.

A regression model is designed to measure the correlation between soil moisture, rainfall and topographical variables. The soil moisture is adopted as a dependent variable, and rainfall, slope, and aspect became independent variables using multiple linear regression. The methodology of modeling allowed determining the leading factors of soil moisture variation across Tamil Nadu. The purpose of the regression analysis was to estimate the relative contribution of each independent variable to the total soil moisture levels. Through these relationships, the study aimed at gaining a better insight into how the interaction between the rain and the topography contributed to the dynamics of soil moisture across the area. Machine Learning models used in the study included RF, SVM, GB, XGBoost, LSTM-ALO, LSTM-INFO, RVFL-EROA, ANN-ERUN, and RVM-IMRFO to examine the correlation among the dependent and independent variables.

### Random forest (RF)

Random Forest is an ensemble learning algorithm that builds multiple decision trees during training and combines their outputs to improve predictive accuracy. In classification tasks it returns the majority vote (mode) of the individual trees, and in regression tasks it returns the average (mean) of their predictions^36^. When constructing each tree, one uses a random subsample size of the training data (with replacement), and a random subsample size of features per split. This randomness is useful in minimizing overfitting and improves the ability of the model to make predictions on the unobserved data. It is especially powerful in the processing of large high-dimensional data and resistant to noise and overfitting.

### Linear Regression

Linear regression refers to a statistical tool that is employed to explain the relationship between a dependent variable and one or more independent ones by modelling a straight line that best fits the observed data^37^. In the simplest version, which is referred to as simple linear regression, the model employs only one independent variable to predict the result. In cases where there are more than two independent variables the method is known as multiple linear regression. Linear models are based on the assumption of their linear association between inputs and outputs and can deliver interpretability and understanding of how separate features affect predictions.

### Support vector machine (SVM)

Support Vector Machine (SVM) is a powerful supervised learning algorithm that is extensively applied in classification as well as regression problems, and is well known to work effectively in high-dimensional spaces^38^. It works by determining the ideal hyperplane that separates the points of the two classes to maximize the margin to increase the overall classification rate and generalization. SVM is suitable when dealing with high-dimensional data and can provide the ability to model complex nonlinear relationships by means of kernel functions.

### Gradient boosting (GB)

Gradient Boosting is an ensemble learning algorithm that constructs models in a series, where each new model attempts to address residual error that earlier models have produced, thus contributing to better overall performance^39^. It maximizes a loss functional by including weak learners (usually decision trees) in stages. Gradient Boosting is capable of operating with complex data and on regression problems especially, but it can be easily overfitted when not tuned correctly.

### XGBoost (Extreme gradient Boosting)

Gradient boosting has been optimized to speed up and perform better on large datasets using XGBoost. It uses regularization methods to minimize overfitting and can process large datasets in parallel, which is suitable in large datasets^39^. XGBoost is a powerful predictive modeler that is capable of modeling complicated relationships and with absent information as it does not tend to exclude it.

### LSTM-ALO (Long Short-Term Memory - Ant Lion Optimizer)

The LSTM (Long Short-Term Memory) networks are used to extract long-term dependencies in time series. Ant Lion Optimizer (ALO) is applied in the LSTM-ALO hybrid to adjust the LSTM parameters (weights, hyperparameters, etc.) to improve performance. ALO is a metaheuristic which is inspired by nature and optimizes the LSTM settings on the fly. When the LSTM is optimized by using ALO, the model yields greater forecasting accuracy^40^.

### LSTM optimized with INFO algorithm (LSTM-INFO)

This methodology represents a combination of an LSTM network and the INFO algorithm, a weighted mean-of-VEs optimizer. INFO uses the parameters of LSTM to refine the learning process to find the best nonlinear and seasonal trends in the data through a series of population-based searches. Practically, LSTM-INFO has been found to minimise forecast errors and enhance correlations over a LSTM^31^.

### Random vector functional link optimized using enhanced reptile optimization algorithm (RVFL-EROA)

Random Vector Functional Link (RVFL) networks are single-layer feedforward models with random hidden nodes and direct input-to-output links, allowing very fast training. In RVFL-EROA, an Enhanced Remora Optimization Algorithm (EROA) is used to tune the RVFL’s weights and biases. EROA is an improved variant of the Remora Optimization Algorithm (it adds strategies like adaptive dynamic probability and Lévy-flight search)^41^. By applying EROA to optimize the RVFL, the hybrid model attains more accurate forecasts.

### Artificial neural network optimized via elite reptile updating network (ANN-ERUN)

ANN-ERUN is a hybrid model that integrates the traditional Artificial Neural Network (ANN) architecture with a metaheuristic optimization algorithm known as the Elite Reptile Updating Network (ERUN)^42^. This optimizer is inspired by the hunting, tracking, and adaptive behavior of reptiles in nature. The ERUN algorithm improves upon the standard Reptile Search Algorithm (RSA) by introducing elite updating and adaptive position-updating strategies, which significantly enhance both exploration and exploitation.

### Relevance vector machine tuned with improved Manta-Ray foraging optimization (RVM-IMRFO)

Relevance Vector Machine (RVM) is a sparse Bayesian regression model that is optimized using the Improved Manta-Ray Foraging Optimization (IMRFO) algorithm. IMRFO is a variant of the Manta-Ray Foraging Optimization enhanced with opposition-based learning to improve global search convergence. In RVM-IMRFO, IMRFO tunes the RVM’s kernel parameters^43^.

### Model setup

Step 1: Prepare the dataset by merging soil moisture and rainfall data, ensuring all variables are at the same resolution (0.25-degree).

Step 2: Define the dependent variable (soil moisture) and independent variables (rainfall and topographical features).

Step 3: Split the data into training (70%) and testing (30%) sets, maintaining the distribution of soil moisture levels.

Step 4: Initialise the model (including RF, SVM, GB, XGBoost, LSTM-ALO, LSTM-INFO, RVFL-EROA, ANN-ERUN, and RVM-IMRFO) with the training datasets and define the model parameters.

Step 5: Train the model on the training dataset, and generate predictions for the testing set.

Step 6: Evaluate model performance using metrics such as RMSE, MAE, NSE, KGE and CA.

The models have their own advantages, with RF being the most robust to noise and having a feature importance perspective, which makes it appropriate to describe complex relationships between climatic and topographical factors. Linear Model is simple and easy to understand, and can be adopted to make comparisons with performance. SVM is better in high dimensional space which makes it effective in modeling non-linear association without overfitting. GB has a reputation of high predictive power and flexibility to optimize loss functions which enables it to be flexible to a particular objective involving soil moisture prediction. The choice of XGBoost is based on efficiency and state of the art of working with big data and also has regularization features that increase the generalizability. Additionally, hybrid models such as LSTM-ALO and LSTM-INFO combine the strengths of traditional machine learning algorithms with deep learning, allowing for improved accuracy and interpretability. RVFL-EROA, RVM-IMRFO and ANN-ERUN, on the other hand, are designed to enhance model robustness through regularization and feature optimization, ensuring better performance in capturing intricate patterns in environmental data.

### Performance metrics

Six dominating indicators are used to assess the performance of the model: Mean Absolute Error (MAE), Root Mean Square Error (RMSE), Nash-Sutcliffe Efficiency (NSE), Kling-Gupta Efficiency (KGE), Coefficient of Determination (R^2^)^44^ and Combined Accuracy^45^.

1 $$\:MAE=\frac{1}{n}\sum\:_{i=1}^{n}{|Y}_{i}^{obs}-{Y}_{i}^{sim}|$$

2 $$\:\text{R}\text{M}\text{S}\text{E}=\sqrt{\frac{1}{\text{n}}\sum\:_{i=1}^{n}{\left({Y}_{i}^{obs}-{Y}_{i}^{sim}\right)}^{2}}$$

3 $$\:\text{N}\text{S}\text{E}=1-\left[\frac{\sum\:_{i=1}^{n}\:\:{\left({Y}_{i}^{obs}-{Y}_{i}^{sim}\right)}^{2}}{\sum\:_{i=1}^{n}\:{\left({Y}_{i}^{obs}-{Y}^{mean}\right)}^{2}}\right]$$

4 $$\:\text{K}\text{G}\text{E}=1-\sqrt{{\left({\rm\:Y}-1\right)}^{2}+{\left(\alpha\:-1\right)}^{2}+{\left(\beta\:-1\right)}^{2}}$$

5 $$\:{R}^{2}=1-\frac{\sum\:_{i=1}^{n}{({Y}_{i}^{obs}-{Y}_{i}^{sim})}^{2}}{\sum\:_{i=1}^{n}{({Y}_{i}^{obs}-\overline{{Y}^{obs}})}^{2}}$$

6 $$\:CA=0.33\:(RMSE+MAE+(1-{R}^{2})$$

where, $\:{{Y}_{i}}^{obs}$ is the $\:i$^th^ observation for the constituent being evaluated, $\:{{Y}_{i}}^{sim}$ is the $\:i$^th^ simulated value for the constituent being evaluated, $\:{Y}^{\text{mean\:}}$ is the mean of observed data for the evaluated constituent, and $\:n$ is the total number of observations, $\:{\rm\:Y}$ is the linear correlation between observations and simulations, $\:\alpha\:$ is a measure of the variability, and $\:\:\beta\:$ is a measure of bias. These evaluation metrics are essential in assessing the performance of predictive models and in quantifying the accuracy of the models.

## Results

By following the above-mentioned methodology, soil moisture prediction over Tamil Nadu was trained using the input variables. A detailed summary of the performance of each machine learning model is provided in Table 1, which applies six evaluation metrics, namely: RMSE^46^, MAE, NSE, KGE, R^2^ and CA. All of these measures indicate the extent to which the predicted values match the observed values of soil moisture, and how well each model describes the variability and effectiveness of prediction.

**Table 1:** Model performance evaluation.

Model	RMSE	MAE	NSE	KGE	*R* ^2^	CA
Random Forest	0.019	0.012	0.983	0.989	0.983	0.016
Linear Regression	0.110	0.084	0.435	0.520	0.435	0.250
SVM	0.065	0.057	0.802	0.870	0.802	0.110
Gradient Boosting	0.038	0.030	0.934	0.885	0.934	0.044
XGBoost	0.018	0.012	0.984	0.989	0.984	0.015
ANN (Dense)	0.021	0.015	0.980	0.973	0.980	0.018
LSTM-ALO	0.155	0.132	-1.181	0.080	-1.181	0.814
LSTM-INFO	0.122	0.084	-0.351	0.443	-0.351	0.514
RVFL-EROA	0.045	0.033	0.904	0.931	0.904	0.057
ANN-ERUN	0.020	0.014	0.981	0.970	0.981	0.018
RVM-IMRFO	0.052	0.036	0.875	0.913	0.875	0.070

Random Forest (RF) and XGBoost (XGB) were the only algorithms with almost identical and better results, having the lowest RMSE (0.019 and 0.018 m³/m³) and MAE (0.012 m³/m³) values, and highest NSE and KGE (> 0.98 and 0.98, respectively). Exceptional precision was also confirmed by their combined accuracy (CA ≈ 0.015–0.016). These models have an ensemble learning framework that allows them to address nonlinear relationships between a variety of hydro-topographic predictors (such as rainfall, elevation, slope, and aspect). The higher XGBoost and Random Forest models performance in this study is due to the capabilities associated with the two classifications in their capacity to capture complex and non-linear relationship, and interactions between hydrological and topographical variables. Rainfall is the most active of the input predictors attributed to its dominance in influencing the soil moisture estimates as it is the most important hydrological driver in monsoon-dominated regions. Topographic features such as slope and aspect, also had a significant effect, as it controlled the surface runoff and surface exposure to sunlight, which in turn influenced the infiltration and evapotranspiration. Ensemble tree-based models like XGBoost and Random Forest were particularly well-suited for this application due to their robustness to noise, ability to model non-linearity, and internal feature selection mechanisms that reduce overfitting. The performance superiority of XGBoost was due to its gradient boosting structure with regularization which provides better generalization, whereas the advantage of Random Forest was on the aspect of bootstrapping and selection of features at random, which implied feature stability in different physiographic areas.

Artificial Neural Network (ANN) and a hybrid variant ANN-ERUN also produced good outcomes (RMSE = 0.020–0.021 m³/m³; NSE ≈ 0.98), which implies that they can learn more complex nonlinear functions at the cost of slightly higher bias than ensemble tree models. Support Vector Machine (SVM) and Gradient Boosting (GB) models, in turn, showed intermediate results (RMSE = 0.038–0.065 m³/m³; NSE = 0.80–0.93), implying that they partially accounted for the variability but were unable to generalize to extreme rainfall conditions and heterogeneous soil than the independent one. The Relevance Vector Machine (RVM-IMRFO) and RVFL-EROA hybrids were equally effective (NSE ≈ 0.87–0.90), which validated their applicability in the case of data-sparse or non-stationary.

Conversely, the Linear Regression (LR) had a weak predictive capacity (RMSE = 0.110 m³/m³; NSE = 0.435), to represent nonlinear correlations of soil moisture with climatic-topographic factors. Likewise, the LSTM-ALO and LSTM-INFO models, although they have the benefit of learning time, produced negative NSE value, and this implies they were worse at predicting compared to the mean predictor. Their high RMSE (0.122–0.155 m³/m³) and high CA (> 0.5) indicate that convergence and overfitting are difficult due to the limited time range of the data. The findings indicate that the ensemble tree-based models, namely, XGBoost and Random Forest are the most likely to work during the prediction of the monsoon-season soil moisture in Tamil Nadu. Their superior performance highlights the importance of capturing nonlinear hydrological interactions in regions characterized by intense rainfall variability and complex terrain.

**Fig. 2:**
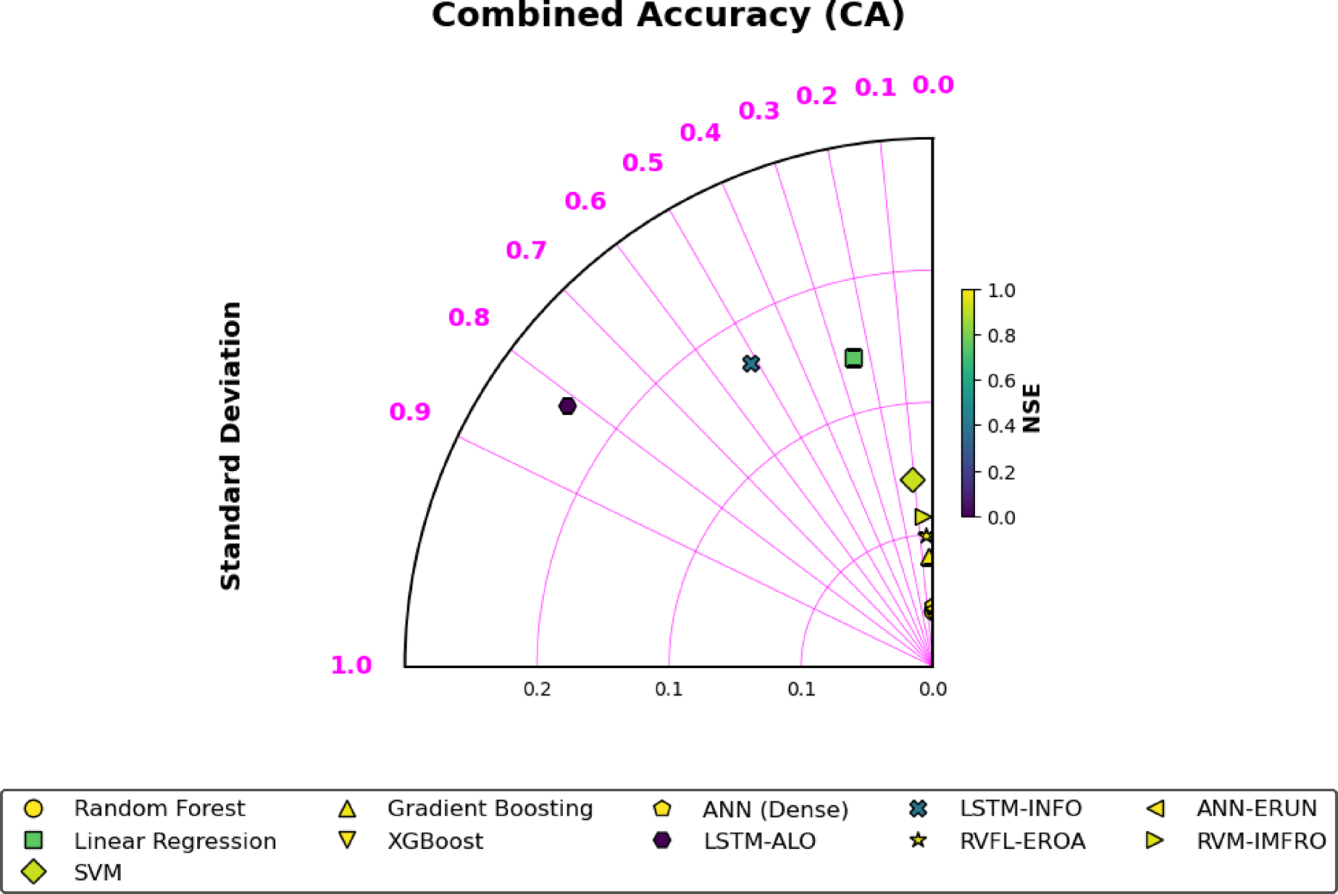
Taylor diagram for comparing NSE and combined accuracy.

Figure 2 (Taylor Diagram) is a comparative visualization of the performance of various machine learning models in estimating the soil moisture in Tamil Nadu. The diagram portrays the capacities of the model to measure the variability (Standard Deviation) in addition to the correlation to the measured soil moisture (Nash-Sutcliffe Efficiency, NSE). The similarity between the Random Forest (RF) and XGBoost (XGB) and the optimal point in terms of predictive accuracy shows that both models are strong in terms of capturing the spatiotemporal variability in soil moisture of the various topographies of Tamil Nadu. Conversely, LSTM-ALO and LSTM-INFO have lower positions relative to the ideal point, a tendency that implies that their performance was comparatively low, and the negative NSE values imply that they outperformed worse than a typical predictor. This outcome underscores the challenges faced by these models in capturing the complex non-linearities inherent in soil moisture dynamics, particularly under varying climatic and topographical conditions. Random Forest and XGBoost are ensemble-based techniques that have proven to be the most stable predictors of soil moisture in areas with complicated topography and a significant amount of rainfall variability, resulting in them being the models of choice when it comes to soil moisture mapping.

Recently, a number of studies have examined ML models in order to analyse and model soil moisture based on rainfall, and topographic or environmental features. Houben et al. (2025) were able to predict field-scale soil moisture on a grassland hillslope with four ML models, including those of random forest (RF), gradient-boosted regression trees (GBRT), support vector regression (SVR), and neural networks with the help of such input variables as terrain characteristics, soil properties and weather conditions. Random Forest and GBRT showed the most impressive results with R² of 0.48–0.69 and RMSE of 0.06–0.10 m³/m³. Similarly, Acharya et al. (2021) examined the soil moisture prediction in agricultural fields based on CART, RF, boosted regression tree (BRT), multiple linear regression (MLR), squared velocity regression (SVR), and artificial neural network (ANN). Weather data, soil properties and crop factors serve as inputs to achieve soil moisture and it is established that Random Forest and BRT yielded best results with RMSE of about 0.045–0.048 m³/m³ (MAE < 0.040 m^3^/m^3^) and correlation r² of about 0.67–0.72 between predicted and measured moisture.

**Fig. 3:**
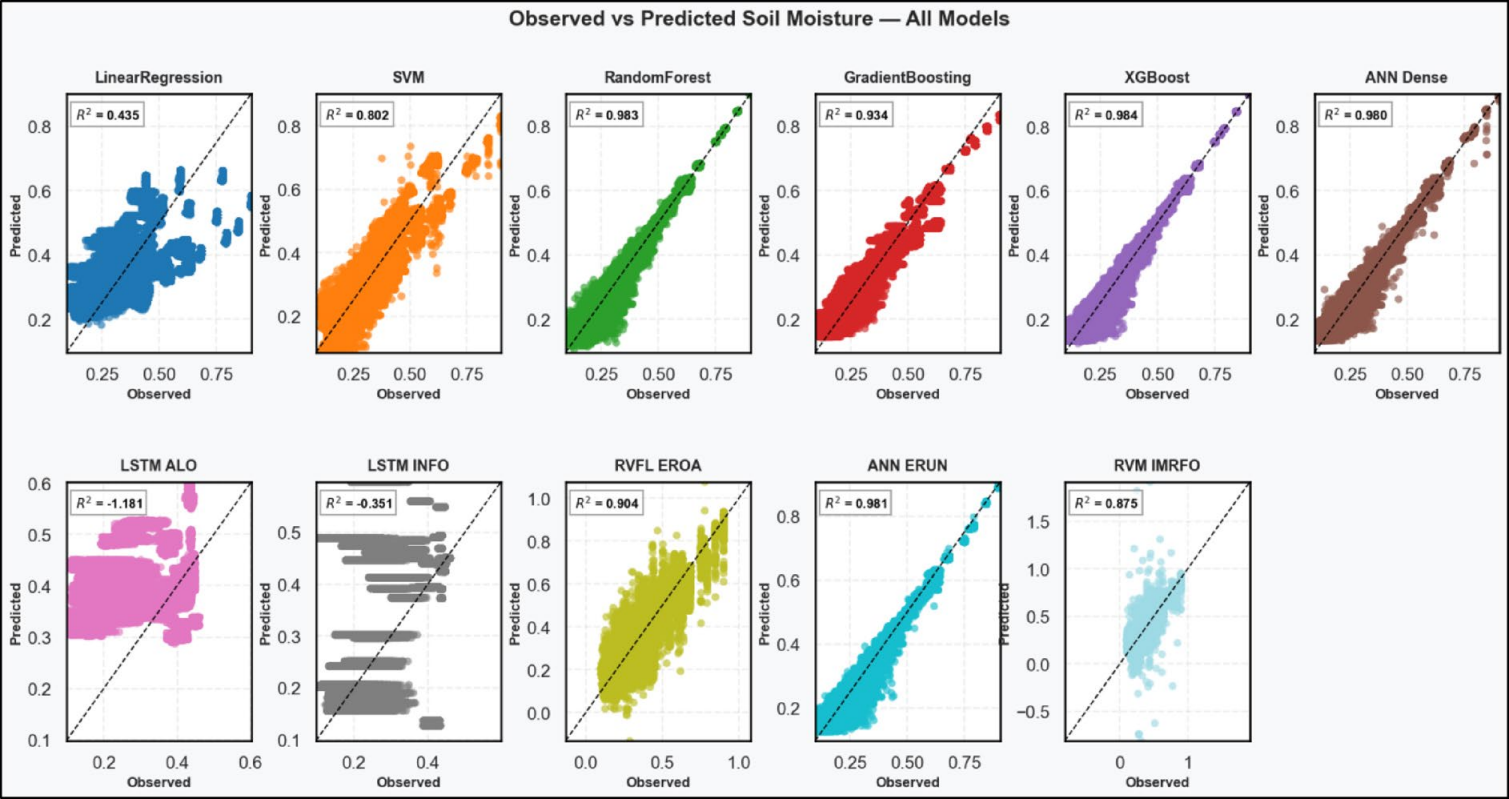
scatter plot comparing the observed and the predicted values.

Scatter plot that compares the observed and the predicted values of each model has been plotted as indicated in Fig. 3. The image offers a straight-forward comparison of the proximity of the predicted values to the measured soil moisture values. The outcomes of the ML analysis to predict soil moisture demonstrate the high degree of differences in the performance of the models, thus pointing out the strengths and weaknesses of the models. Out of all the models, XGBoost and Random Forest appear the most precise with the R² of 0.984 and 0.983, respectively. The forecasts of these models are very near to the 1:1 line of reference with a high density of tight clusters with low dispersion. It implies not just good model fitting, but good generalization at both high and low moisture levels, which is an important property in situations where the moisture regime of the source location, such as monsoonal areas such as Tamil Nadu, are highly seasonal and terrain dependent. They are especially well-equipped to carry out this task due to their capability to learn more complicated nonlinear relationships between rainfall, slope and land characteristics.

ANN ERUN (R² = 0.981) and ANN Dense (R² = 0.980) also provide satisfactory results, but the scatter diagrams demonstrate the existence of slightly broader distribution along the 1:1 line. This implies that while neural networks capture the general pattern quite well, they may exhibit marginally higher variance in predictions, possibly due to overfitting or insufficient representation of edge cases during training. RVFL EROA (R² = 0.904) and RVM IMRFO (R² = 0.875) exhibit fair predictive abilities, but with growing dispersion, especially with higher levels of soil moisture. SVM (R² = 0.802) adheres to this pattern but displays a significant dispersion in the range under observation, showing tendencies to have generalization issues, particularly at the extremes. Gradient Boosting (R² = 0.934) is more effective, however, its scatterplot is more dispersed than RF and XGBoost, perhaps due to the weakness of its ability to capture interaction without enough tuning or regularization. It falls between high and average performance. Linear Regression does not capture the nonlinear dynamics of the soil moisture dynamics with an R² of only 0.435. The scatter is extensive and skewed at each end with a preference to the lower moisture values, which confirms that it cannot be adapted to the complexities of the data.

The LSTM-based results of LSTM ALO (R² = -1.181) and LSTM INFO (R² = -0.351) present the least performance of the models. Both models yield least predictions, detached from any alignment with observed values. Their negative R² scores are negative indicating that they are even worse than mere prediction of the mean and their scatterplots are diffuse clouds that do not seem to have a pattern. These results could be attributed to suboptimal hyperparameter settings, lack of sequential structure in the input data, or insufficient temporal features that render LSTMs unsuitable for this task in their current configuration.

**Fig. 4:**
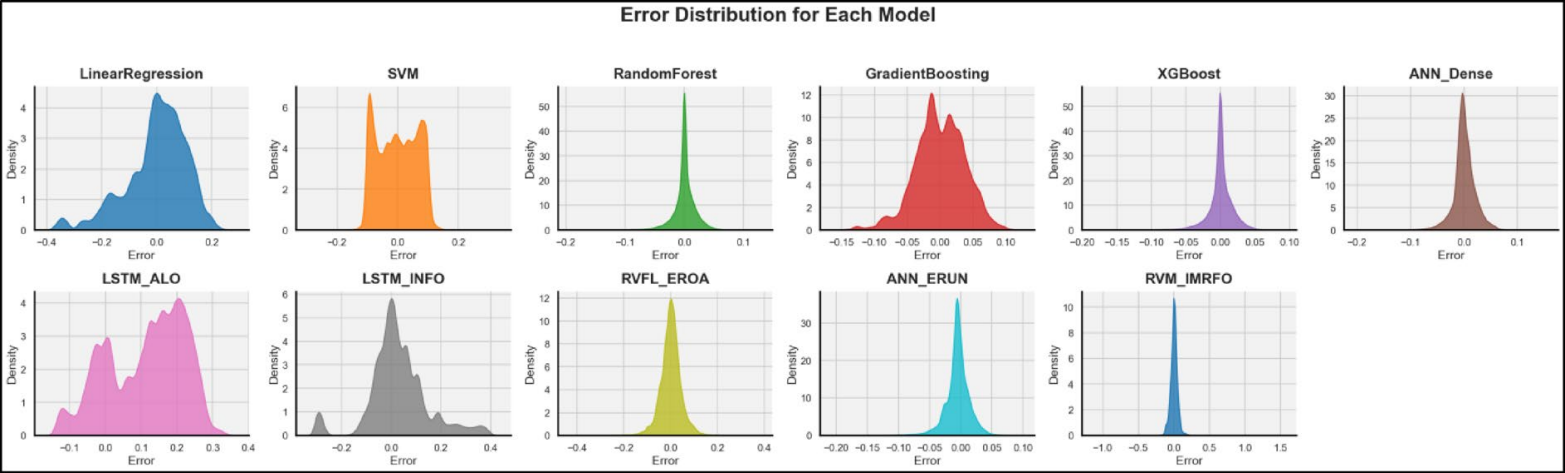
Error distribution plots for the ML models.

The error distribution figures (Fig. 4) of the models also help in strengthening the conclusion made based on the performance measures (RMSE, MAE, NSE, KGE, R^2^ and CA) and scatter plots. The most desirable behavior is shown by Random Forest (RF) and XGBoost (XGB) models, where both distributions are highly concentrated with sharp peaks that are almost centered around zero. The implication of such narrow and symmetric curves is that the two models provide nearly accurate predictions of soil-moisture with minimal bias, or variance, and it is ascertained that the two models provide a high-quality R² and NSE. Similarly compact, zero-centered peaks are also observed in the Artificial Neural Network (ANN-Dense) and ANN-ERUN models and suggest a stable generalization and a low systematic deviation. However, the Support Vector machine (SVM) and LSTM-INFO models have slightly wider and more skewed distribution, indicating moderate variability and slight bias in their residual. This pattern indicates that, although these models capture the nonlinear structure of the data better than simpler regressors, they occasionally over- or under-estimate soil-moisture extremes. The more wider and flatter distribution of the Gradient Boosting (GB) model with longer tails on each side indicates that it has a greater probability of large error especially underestimation, which is consistent with its lower NSE and larger RMSE. Meanwhile, LSTM-ALO model exhibits a two peak error pattern that might be due to multimodal dynamics or lag effects that were not fully modeled in the training period. Although RVFL-EROA and RVM-IMRFO models do not typically generate errors with a large range, the error distribution is moderate in these models, which is a characteristic of an unstable model in complex nonlinear interaction processes. Lastly, Linear Regression (LM) model has a wide, irregular, and left-skewed distribution characterized by high rates of underprediction and its inability to explain nonlinear processes of soil-moisture. Together, these trends confirm that ensemble tree-based and deep-learning models (RF, XGB, ANN-ERUN) do not only outperform the traditional algorithms but also retain a narrower error control range when using them in different soil-moisture contexts, which reinforces their usefulness in the hydro-climatic context.

**Fig. 5:**
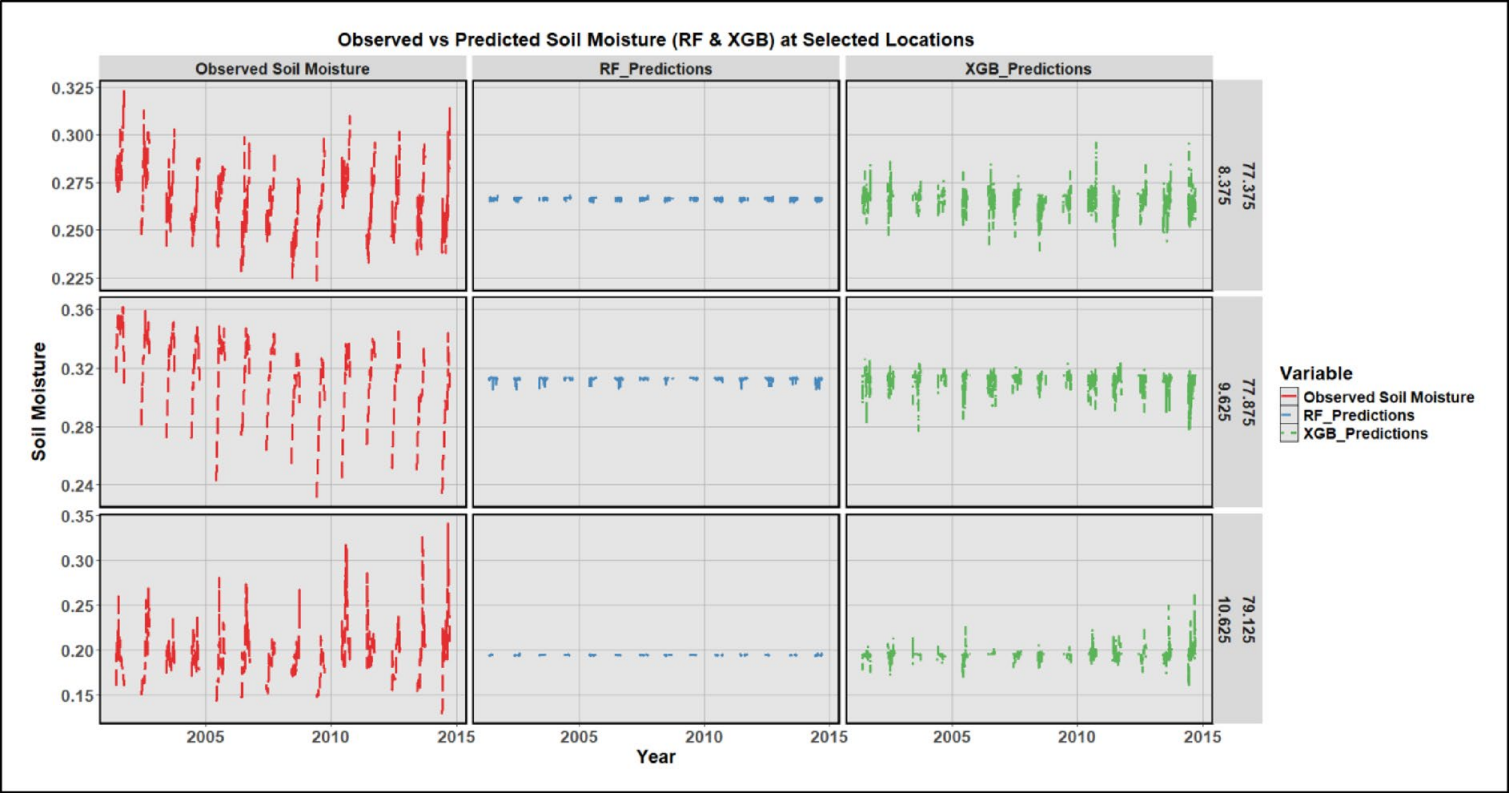
Comparison of soil moisture observations and predictions from various models.

Comparison of the soil moisture produced by the ML models at random locations so as to determine which model fits best. Cosnidering six metrics (RMSE, MAE, NSE, KGE, R^2^, CA) both ensemble trees (XGBoost and RF) significantly prevail, demonstrating capacity to predict soil moisture using rainfall data. XGBoost records the best errors (RMSE = 0.018 m³/m³; MAE = 0.012 m³/m³) and the highest efficiency/skill (NSE = 0.984; KGE = 0.989; R² = 0.984), with Random Forest statistically indistinguishable (RMSE = 0.019; MAE = 0.012; NSE = 0.983; KGE = 0.989; R² = 0.983). Their Combined Accuracy is the lowest in the suite (CA ≈ 0.015–0.016), indicating that multiple independent metrics simultaneously agree these models are highly reliable. Comparing the performance of RF and XGBoost models in relation to soil moisture observation and soil moisture prediction across three random locations (Fig. 5) depicts important observations to the performance of each model. In all three locations, the observed soil moisture shows clear seasonal variability, with sharp peaks during monsoon months, consistent with rainfall-induced recharge and subsequent evapotranspiration losses. This temporal trend is again replicated by the RF predictions with highly consistent predictability and low variance, but with more smoothing between wet and dry conditions suggesting a high predictive consistency but narrower dynamic range than observations. The XGB predictions display a closer alignment with observed patterns, especially in capturing the amplitude and frequency of monsoon peaks. The variability and dispersion of XGB results imply that it is more sensitive to changes in inputs thus allowing it to capture smaller variations in soil moisture. On all three sites, both ensemble models can reproduce the temporal dynamics of soil moisture, but XGBoost is a bit more responsive to the local hydrology of the extremes but the predictions of the Random Forest are smoother and more conservative. The fact that the predicted and observed signals correlate confirms the generalization capability of both models to physiographically dissimilar regions. Notably, model predictions maintain consistency across years, showing that the trained models can effectively reproduce interannual soil-moisture variability despite variations in rainfall intensity and land-surface characteristics. overall, Fig. 5 highlights the strong performance of the ensemble learning models to predict the soil moisture within the monsoon-affected climatic conditions. Random Forest model is better at prediction noise reduction and maintaining time consistency whereas XGBoost is better at describing and predicting steep temporal gradients and rain-driven peaks, confirming its high-quality performance indicators in Table 1.

**Fig. 6:**
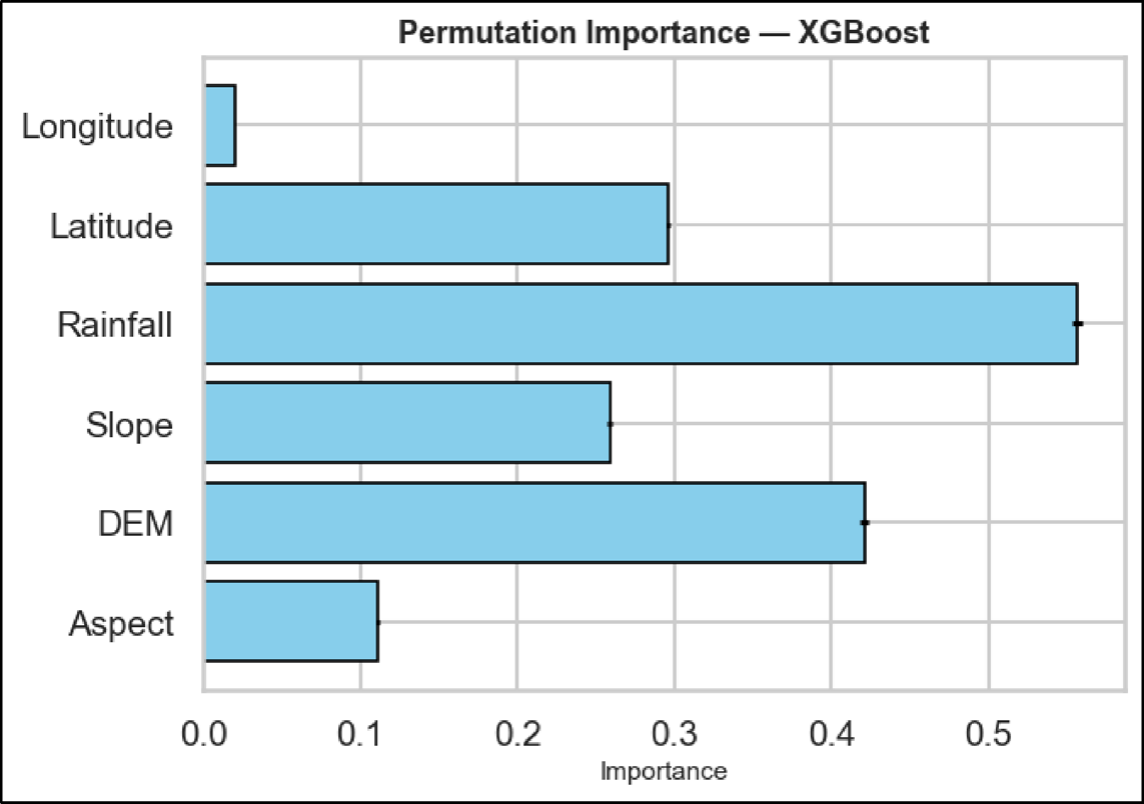
Permutation importance of XGBoost model.

Permutation importance is a model agnostic approach of measuring the contribution of each feature to the performance of a trained model. Figure 6 depicts the permutation importance of all the parameters that were applied to build the XGBoost model. The rainfall is found to be the most dominant, as the monsoonal precipitation is the main source of soil moisture and its variability determines the moisture deficits directly. Topography, elevation (DEM) and slope are the obvious secondary controls. The elevation also affects the moisture amount and is said to trap orographic rain and sustains colder temperatures (lowers evapotranspiration) and slope gradient determines the ratio between surface water and sub-surface water (steeper slopes reduce water retention). Latitude has moderate significance, probably acting as a proxy of extensive northsouth climatic gradients in insolation and monsoon intensity. Lastly, the insignificant role played by aspect and longitude; the heavy saturating rains in this humid monsoon climate outweigh the effect that slope direction (aspect), and the close eastwest gap between the study area requires that longitude does not relate to a meaningful atmospheric variation. Therefore, the model proves that monsoon rains are the major control, and terrain is the major modulating factor.

Although the predictive performance is high in this study, there are some limitations that limit the generalizability and strength. To begin with, the model does not utilize the other important input variables, such as vegetation indices (e.g., NDVI/EVI), land use/land cover, soil texture, evapotranspiration, and surface temperature, which, however, have a significant impact on the soil moisture dynamics. The inclusion of these other variables may lead to better model generalisation and accuracy in a wide variety of landscapes. Second, the models trained on non-temporal non-seasonal data without taking temporal relationships into consideration. The memory effects of soil moisture are affected by the patterns of antecedent rainfall and evapotranspiration. In this way, temporal dynamics might be more accurately represented by the use of lagged rainfall or deep learning models, e.g., recurrent neural networks (RNNs) or long short-term memory (LSTM) networks. Thirdly, the temporal scope of the study is limited to the monsoon months (June–September) from 2001 to 2014, which may not reflect recent land use changes or interannual climate variability. Extending the analysis to other seasons and more recent years using updated datasets would improve relevance for year-round applications. Moreover, the reference soil moisture dataset derived from Nayak et al. (2018) is based on a land data assimilation system, which may introduce model-related biases, particularly in the absence of validation with in-situ observations. He reported the validation statistics showing a correlation coefficient (R) ranging from 0.5 to 0.89, RMSE from 0.024 to 0.131 m³/m³, and bias from − 0.065 to 0.011 m³/m³ across various in-situ sites. While these metrics suggest reasonable overall accuracy, they also highlight spatial and depth-wise variability, and inherent uncertainty. This could affect the reliability of the training data and the performance metrics reported. Additionally, the spatial transferability of the model was not explicitly tested across different agro-climatic zones in Tamil Nadu, raising concerns about potential overfitting to dominant regions. Implementing spatial cross-validation techniques and testing model performance in varied topographic and climatic zones could help address this. Finally, while the study proves the efficacy of machine learning for soil moisture prediction, it does not outline a pathway for real-time or operational deployment. Future research should focus on integrating real-time data streams, automating model updates, and developing interactive platforms for stakeholders in agriculture and water management. Addressing these limitations would not only enhance the scientific rigor of the model but also improve its practical utility in environmental decision-making.

## Discussions

In this study, an in-depth examination of machine learning models to estimate soil moisture in Tamil Nadu was conducted, with rain and topographical data. Out of 11 models that were tested, the ensemble methods, namely XGBoost and Random Forest (RF), displayed an impressive performance, being superior to the other models in all measures. Both XGBoost and RF had the lowest values of RMSE (0.018 and 0.019 m³/m³, respectively) and the highest Nash-Sutcliffe Efficiency (NSE) and Kling-Gupta Efficiency (KGE) (> 0.98), reinforcing their suitability for modeling complex, non-linear interactions inherent in soil moisture dynamics. These models, through their ensemble approach, effectively captured the intricate dependencies between rainfall and topographical features, making them ideal for this heterogeneous landscape. This finding aligns with previous studies, such as those by Houben et al. (2025) and Acharya et al. (2021), which highlighted the success of Random Forest and gradient-boosted models for soil moisture predictions in diverse environmental contexts. The superior performance of XGBoost was attributed to its gradient-boosting framework, which incorporates regularization techniques to minimize overfitting, while Random Forest’s strength lies in its bootstrapping mechanism and feature selection process, ensuring robustness across varied topographies.

The Artificial Neural Network (ANN) and its hybrid version, ANN-ERUN, also delivered competitive results (RMSE ≈ 0.020 m³/m³, NSE ≈ 0.980), reflecting their ability to model complex non-linear relationships. However, their performance was slightly inferior to the ensemble methods, possibly due to higher biases or slight overfitting tendencies. On the other hand, models like Support Vector Machine (SVM), Gradient Boosting (GB), and the hybrid approaches, RVFL-EROA and RVM-IMRFO, exhibited moderate performance, with RMSE values ranging from 0.038 to 0.065 m³/m³. These models captured part of the variability but struggled with generalization, particularly under extreme rainfall or topographically diverse conditions. Linear Regression (LR), by contrast, performed poorly, with RMSE of 0.110 m³/m³ and NSE of 0.435, highlighting its inability to account for the complex, non-linear dependencies between the input variables and soil moisture.

The Taylor Diagram (Fig. 2) visually confirmed the superior predictive accuracy of XGBoost and Random Forest. Both models were closest to the ideal point, demonstrating their exceptional ability to match both the variability and the correlation of observed soil moisture. The scatter plots further emphasized this, with XGBoost and RF showing narrow distributions around the 1:1 line, indicating their strong alignment with observed values. LSTM-based models, particularly LSTM-ALO and LSTM-INFO, were positioned farther from the ideal point, with negative NSE values, underscoring their inability to perform better than the mean predictor. This result is indicative of challenges faced by these models in capturing the non-linear nature of soil moisture dynamics, especially under diverse climatic conditions.

Despite these promising results, the study has certain limitations. The reliance on a limited set of predictors primarily rainfall, slope, and aspect may have restricted the model’s ability to fully account for the multifaceted drivers of soil moisture. Future studies should consider incorporating additional variables, such as vegetation indices (e.g., NDVI), soil texture, and evapotranspiration, to improve the models’ performance across varied landscapes. Furthermore, the models were trained using data from the monsoon season only (June–September), and their generalizability over other seasons and years remains untested. It is also important to note that the gridded soil moisture dataset used for training was derived from a land data assimilation system, which may introduce some level of model-related bias. Validating this dataset with ground-based measurements could enhance the reliability of the results. The spatial transferability of the models across Tamil Nadu’s agro-climatic zones was also not tested, and this could be a potential avenue for future research direction. Lastly, integrating temporal dependencies and lagged variables through architectures like LSTMs or Recurrent Neural Networks (RNNs) could improve the modeling of soil moisture dynamics over time.

## Conclusions

The study has managed to assess the performance of different machine learning models in soil moisture predictions and reveal the significant variation in the effectiveness of the models. XGBoost became the most accurate model of the examined models and was successful in capturing the complex, non-linear relationships that the dynamics of the soil moisture are associated with. The findings showed that ensemble techniques, especially XGBoost and Random Forest, are more effective when dealing with the complexity of environmental data compared to less complex methods such as Linear Regression that could not adapt well to the non-linear behavior of the soil moisture change. The overall performance metrics and visual comparisons additionally support the fact that superior modeling methods are required in environmental studies.

There are some important directions that can be investigated in order to improve the accuracy of soil moisture predictions. The inclusion of other variables, including historical weather (temperature, humidity and wind speed), land cover type and soil characteristics (e.g. texture and permeability) may greatly enhance predictive ability of the models. The additions would contribute to a better modeling of underlying physical processes and increase the model application to the variety of agro-climatic regions. The introduction of stacked ensemble models is a potential solution. Stacking is the process of combining the predictions of a set of different algorithms i.e. XGBoost, Random Forest, Support Vector Machines, etc. to create a stronger meta-model. The method is capable of harmonizing the advantages and disadvantages of single models and produce a better generalizability and accuracy in the diverse spatial and temporal conditions. The use of deep learning methods, especially Recurrent Neural Networks (RNNs) and Long Short-Term Memory (LSTM) networks, has great potential. These architectures are specifically crafted to understand time based dependencies of sequential data and are, therefore, best formulated to represent the memory effects and seasonal lags of soil moisture dynamics.

Also, to enhance the credibility and reliability of soil moisture predictions using ML, future research should focus on validation through ground-based in-situ measurements. The spatial transferability of models is also suggested to be investigated through testing and crossing the models between various agro-climatic zones and spatial cross-validation methods. Moreover, they can be implemented in real-time environments with automated data feeds and user-friendly control panels to assist in their integration in operational environments to support precision agriculture, drought early warning, and water resource planning. By exploring these enhancements, enriching the predictor space, adopting ensemble and deep learning frameworks, validating with in-situ data, testing for spatial generalizability, and enabling real-time deployment, future research can significantly advance the precision and impact of soil moisture modeling, enabling data-driven decision-making in agricultural and environmental systems.

## Data Availability

The dataset utilized/analyzed during the current study will be available from the corresponding author upon request.

## References

[1] Settu, P. & Ramaiah, M. Estimation of Sentinel-1 derived soil moisture using modified Dubois model. *Environ. Dev. Sustain.*10.1007/s10668-024-05460-1 (2024).

[2] Mondal, S. & Mishra, A. Quantifying the precipitation, evapotranspiration, and soil moisture network’s interaction over global land surface hydrological cycle. *Water Resour. Res.* 60, eWR034861 (2024). (2023).

[3] Yang, X. et al. Differential responses of soil CO2 dynamics along soil depth to rainfall patterns in the Chinese loess plateau. *Agric. Ecosyst. Environ.* **378**, 109306 (2025).

[4] McColl, K. A. et al. The global distribution and dynamics of surface soil moisture. *Nat. Geosci.* **10**, 100–104 (2017).

[5] Chatterjee, S., Desai, A. R., Zhu, J., Townsend, P. A. & Huang, J. Soil moisture as an essential component for delineating and forecasting agricultural rather than meteorological drought. *Remote Sens. Environ.* **269**, 112833 (2022).

[6] Kang, J., Peng, Y. & Xu, W. Crop root responses to drought stress: molecular Mechanisms, nutrient Regulations, and interactions with microorganisms in the rhizosphere. *Int J. Mol. Sci* **23**, (2022). 10.3390/ijms23169310 PMC9409098 36012575

[7] Sundaram, S., Devaraj, S. & Yarrakula, K. Modeling, mapping and analysis of urban floods in India—a review on Geospatial methodologies. *Environ Sci. Pollut Res* **2**, (2021). 10.1007/s11356-021-16747-5 34626336

[8] Green, J. K. et al. Large influence of soil moisture on long-term terrestrial carbon uptake. *Nature* **565**, 476–479 (2019). 30675043 10.1038/s41586-018-0848-x PMC6355256

[9] Geertsema, M. & Alcántara-Ayala, I. Mountain landslides: an overview of common types and future impacts. in Montology Palimpsest: A Primer of Mountain Geographies (ed Sarmiento, F. O.) 187–209 (Springer International Publishing, Cham, doi:10.1007/978-3-031-13298-8_11. (2022).

[10] Mina, U., Dimri, A. P. & Farswan, S. Forest fires and climate attributes interact in central himalayas: an overview and assessment. *Fire Ecol.* **19**, 14 (2023).

[11] Fan, Y. et al. A critical review for Real-Time continuous soil monitoring: Advantages, Challenges, and perspectives. *Environ. Sci. Technol.* **56**, 13546–13564 (2022). 36121207 10.1021/acs.est.2c03562

[12] Tavakol, A., McDonough, K. R., Rahmani, V., Hutchinson, S. L. & Hutchinson, J. M. The soil moisture data bank: the ground-based, model-based, and satellite-based soil moisture data. *Remote Sens. Appl. Soc. Environ.* **24**, 100649 (2021).

[13] Cooper, J. D. Gravimetric method. *Soil. Water Meas.* 26–42. 10.1002/9781119106043.ch6 (2016).

[14] Persico, R., Cataldo, A. & De Benedetto, E. Chapter 3 - Time-domain reflectometry: Current uses and new possibilities. in (eds. Persico, R., Piro, S. & Linford, N. B. T.-I. in N.-S. G.) 59–96Elsevier, (2019). 10.1016/B978-0-12-812429-1.00003-9

[15] Suresh, D. & Yarrakula, K. InSAR based deformation mapping of earthquake using Sentinel 1A imagery. *Geocarto Int.* 1–10. 10.1080/10106049.2018.1544289 (2019).

[16] Devaraj, S. & Yarrakula, K. Assessment of topographical and atmospheric errors in Sentinel 1 derived DInSAR. *Geocarto Int.* 1–17. 10.1080/10106049.2020.1822926 (2020).

[17] Sundaram, S., Devaraj, S. & Yarrakula, K. Mapping and assessing Spatial extent of floods from multitemporal synthetic aperture radar images: a case study over Adyar watershed, India. *Environ. Sci. Pollut Res.* **30**, 63006–63021 (2023). 10.1007/s11356-023-26467-7 36952156

[18] Suresh, D. et al. Morphometric analysis for identification of groundwater recharge zones: A case study of Neyyar river basin. *Indian J. Geo-Mar. Sci.* **47**, 1969–1979 (2018).

[19] Cui, H. et al. Evaluation and analysis of AMSR-2, SMOS, and SMAP soil moisture products in the Genhe area of China. *J. Geophys. Res. Atmos.* **122**, 8650–8666 (2017).

[20] Wang, Q. et al. A comprehensive review of spatial-temporal-spectral information reconstruction techniques. *Sci. Remote Sens.* **8**, 100102 (2023).

[21] Sundaram, S., Devaraj, S. & Yarrakula, K. Modeling, mapping and analysis of urban floods in India—a review on Geospatial methodologies. *Environ. Sci. Pollut Res.* **28**, 67940–67956 (2021). 10.1007/s11356-021-16747-5 34626336

[22] Sivalingam, S., Murugesan, G. P., Dhulipala, K., Kulkarni, A. V. & Devaraj, S. Temporal fluctuations of Siachen glacier velocity: a repeat pass Sar interferometry based approach. *Geocarto Int.* **37**, 4888–4910 (2022).

[23] Abbes, A., Ben, Jarray, N. & Farah, I. R. Advances in remote sensing based soil moisture retrieval: applications, techniques, scales and challenges for combining machine learning and physical models. *Artif. Intell. Rev.* **57**, 224 (2024).

[24] Petropoulos, G. P., Ireland, G. & Barrett, B. Surface soil moisture retrievals from remote sensing: current status, products & future trends. *Phys. Chem. Earth Parts A/B/C*. **83–84**, 36–56 (2015).

[25] Dash, S. S., Sahoo, B. & Raghuwanshi, N. S. How reliable are the evapotranspiration estimates by soil and water assessment tool (SWAT) and variable infiltration capacity (VIC) models for catchment-scale drought assessment and irrigation planning? *J. Hydrol.* **592**, 125838 (2021).

[26] Sehler, R., Li, J., Reager, J. T. & Ye, H. Investigating relationship between soil moisture and precipitation globally using remote sensing observations. *J. Contemp. Water Res. Educ.* **168**, 106–118 (2019).

[27] Maia, R. F., Lurbe, C. B. & Hornbuckle, J. Machine learning approach to estimate soil matric potential in the plant root zone based on remote sensing data. *Front. Plant. Sci.* **13**, 931491 (2022). 36046589 10.3389/fpls.2022.931491 PMC9420971

[28] Wang, S., Li, R., Wu, Y. & Wang, W. Estimation of surface soil moisture by combining a structural equation model and an artificial neural network (SEM-ANN). *Sci. Total Environ.* **876**, 162558 (2023). 36894100 10.1016/j.scitotenv.2023.162558

[29] Adab, H., Morbidelli, R., Saltalippi, C., Moradian, M. & Ghalhari, G. A. F. Machine Learning to Estimate Surface Soil Moisture from Remote Sensing Data. *Water* 12, (2020).

[30] Ahmad, S., Kalra, A. & Stephen, H. Estimating soil moisture using remote sensing data: A machine learning approach. *Adv. Water Resour.* **33**, 69–80 (2010).

[31] Ikram, R. M. A. et al. Water temperature prediction using improved deep learning methods through reptile search algorithm and weighted mean of vectors optimizer. *J Mar. Sci. Eng* **11**, (2023).

[32] Pu, S. et al. Research on mobile ultrasonic stratified flow velocity measurement based on machine learning algorithms. *Front Water Volume* **7–2025**, (2025).

[33] Adnan, R. M. et al. Improved random vector functional link network with an enhanced Remora optimization algorithm for predicting monthly streamflow. *J. Hydrol.* **650**, 132496 (2025).

[34] Adnan, R. M. et al. Pan evaporation Estimation by relevance vector machine tuned with new metaheuristic algorithms using limited Climatic data. *Eng Appl. Comput. Fluid Mech* **17**, (2023).

[35] Nayak, H. P. et al. High-resolution gridded soil moisture and soil temperature datasets for the Indian monsoon region. *Sci. Data*. **5**, 180264 (2018). 30457572 10.1038/sdata.2018.264 PMC6244185

[36] Fawagreh, K., Gaber, M. M. & Elyan, E. Random forests: from early developments to recent advancements. *Syst. Sci. Control Eng.* **2**, 602–609 (2014).

[37] Maulud, D. & Abdulazeez, A. M. A review on linear regression comprehensive in machine learning. *J. Appl. Sci. Technol. Trends*. **1**, 140–147 (2020).

[38] Cervantes, J., Garcia-Lamont, F., Rodríguez-Mazahua, L. & Lopez, A. A comprehensive survey on support vector machine classification: Applications, challenges and trends. *Neurocomputing* **408**, 189–215 (2020).

[39] Bentéjac, C., Csörgő, A. & Martínez-Muñoz, G. A comparative analysis of gradient boosting algorithms. *Artif. Intell. Rev.* **54**, 1937–1967 (2021).

[40] Yilmaz, D. & Büyüktahtakın, İ. E. Learning optimal solutions via an LSTM-Optimization framework. *Oper. Res. Forum*. **4**, 48 (2023).

[41] Wang, S., Hussien, A. G., Jia, H., Abualigah, L. & Zheng, R. Enhanced remora optimization algorithm for solving constrained engineering optimization problems. *Mathematics* 10, (2022).

[42] Khatir, A., Capozucca, R., Khatir, S. & Magagnini, E. Cuong-Le, T. Enhancing damage detection using reptile search Algorithm-Optimized neural network and frequency response function. *J. Vib. Eng. Technol.* **13**, 88 (2025).

[43] Liao, Y., Zhao, W. & Wang, L. Improved manta ray foraging optimization for parameters identification of magnetorheological dampers. *Mathematics* 9, (2021).

[44] Moriasi, D. N. et al. Model evaluation guidelines for systematic quantification of accuracy in watershed simulations. *Am. Soc. Agric. Biol. Eng.* **50**, 885–900 (2007).

[45] Adnan, R. M. et al. Daily streamflow prediction using optimally pruned extreme learning machine. *J. Hydrol.* **577**, 123981 (2019).

[46] Houben, T., Ebeling, P., Khurana, S., Schmid, J. S. & Boog, J. Machine-learning based Spatiotemporal prediction of soil moisture in a grassland hillslope. *Vadose Zo J* **24**, (2025).

